# Alcohol intoxication at Swedish football matches: A study using biological sampling to assess blood alcohol concentration levels among spectators

**DOI:** 10.1371/journal.pone.0188284

**Published:** 2017-11-20

**Authors:** Natalie Durbeej, Tobias H. Elgán, Camilla Jalling, Johanna Gripenberg

**Affiliations:** STAD, Centre for Psychiatry Research, Department of Clinical Neuroscience, Karolinska Institutet & Stockholm Health Care Services, Stockholm County Council, Stockholm, Sweden; Central Queensland University, AUSTRALIA

## Abstract

Alcohol use and alcohol-related problems, including accidents, vandalism and violence, at sporting events are of increased concern in Sweden and other countries. The relationship between alcohol use and violence has been established and can be explained by the level of intoxication. Given the occurrence of alcohol use and alcohol-related problems at sporting events, research has assessed intoxication levels measured through biological sampling among spectators. This cross-sectional study aimed to assess the level of alcohol intoxication among spectators at football matches in the Swedish Premier Football League. Spectators were randomly selected and invited to participate in the study. Alcohol intoxication was measured with a breath analyser for Blood Alcohol Concentration levels, and data on gender, age, and recent alcohol use were gathered through a face-to-face interview. Blood Alcohol Concentration samples from 4420 spectators were collected. Almost half (46.8%) had a positive Blood Alcohol Concentration level, with a mean value of 0.063%, while 8.9% had a Blood Alcohol Concentration level ≥ 0.1%, with a mean value of 0.135%. Factors that predicted a higher Blood Alcohol Concentration level included male gender (*p =* 0.005), lower age (*p <* 0.001), attending a local derby (*p* < 0.001), alcohol use prior to having entered the arena (*p <* 0.001), attending a weekend match (*p* < 0.001), and being a spectator at supporter sections (*p <* 0.001). About half of all spectators at football matches in the Swedish Premier Football League drink alcohol in conjunction with the match. Approximately one tenth have a high level of alcohol intoxication.

## Introduction

The relationship between alcohol and alcohol-related problems such as violence has been established [1–3], and can be explained by factors such as the amount of alcohol consumed and the level of intoxication [4, 5]. There are several negative consequences of alcohol use, both for the individual and society, including psychological and physical suffering as well as economic and public health burdens [6]. During the last decade, there has been increased attention to and research on the negative impact of alcohol consumption on other people, i.e., alcohol’s harm to others.

In recent years, there has been an ongoing debate in both international and Swedish media about the increasing problems of alcohol use at sporting events. Alcohol consumption is often a focal point at these events, and although there are spectators who drink heavily and do not engage in violent behaviour e.g. Danish football supporters known as “Roligans” [7], the propensity for alcohol-related problems, including accidents, vandalism, and violence at sporting events is rather high [8]. The problems have been reported in research [8–11], as has the association between alcohol use and violence among spectators at sporting events [12–14].

Although some studies have demonstrated efforts to reduce alcohol use and alcohol-related problems at sporting events [15, 16], it has also been shown that sport stadiums have a high likelihood of serving alcohol to obviously intoxicated patrons [17]. The matter was highlighted during the 2016 European Football Championship in France, where the French government decided to ban all alcohol sales, on and off premises, and around football stadiums on match days, due to the high number of alcohol-related incidents associated with this event. In Sweden, the problem has been stressed among Swedish policymakers, authorities and key stakeholders, who demand reductions of violence and other alcohol-related problems at sporting events. Similar concerns have been expressed in other countries.

Given the occurrence of alcohol use and alcohol-related problems at sporting events, research has assessed intoxication levels through biological sampling in these settings, for instance at football, baseball [18–20] and cricket matches [21]. For example, studies from the US have measured Blood Alcohol Concentration (BAC) levels among spectators at baseball and American football matches and shown that approximately 40% tested positive for alcohol use, and that the BAC levels ranged between 0.005 and 0.217%, with a mean value of 0.057% among those who had been drinking alcohol [18,19]. Also, spectators who were under the age of 35 were more likely to have a positive BAC level compared with other spectators. Moreover, another US study measuring intoxication levels among college football fans showed that the average BAC level for the entire sample was 0.006%, that 90% of the participants had been drinking alcohol during the match, and that 20% had a BAC level above the legal limit to drive [20]. Additionally, a UK study revealed high levels of binge drinking among spectators at a cricket match and showed that one third exceeded the drink-drive limit according to the BAC assessments [21]. Such results can be considered alarming and might be grounds for intervention efforts to reduce heavy alcohol use and alcohol-related problems at sporting events. To our knowledge, research on intoxication levels measured using BAC levels among spectators at Swedish sporting events is lacking.

One strategy to reduce alcohol use and alcohol-related problems at sporting events is to implement multi-component local interventions, addressing for instance alcohol availability and pricing, stricter enforcement, and training of staff in Responsible Beverage Service (RBS) [15,16,22]. In 1996, our research unit STAD (Stockholm Prevents Alcohol and Drug problems) developed a multi-component community-based RBS prevention programme to reduce heavy alcohol use, intoxication levels, and violence in the nightlife setting [23]. The programme comprised various environmental intervention strategies such as community mobilization, policy work, RBS training of servers, stricter enforcement of existing alcohol laws [24], and was shown to be effective in reducing levels of overserving to under-aged and pseudo-intoxicated patrons [23,25], the number of alcohol-related violent crimes [24,26], societal costs of alcohol-related violent crimes [27], and the number of emergency room visits [28]. Overall, several studies have demonstrated positive effects of RBS programmes in the community, both in Sweden and other countries (e.g., [29,30]).

Given the concerns related to alcohol use and violence at sporting events, STAD has initiated a novel research project aiming to reduce intoxication levels and alcohol-related problems at these events [31]. Since alcohol use is particularly common during football matches in Sweden [32], football arenas serve as the primary setting. The current study is a part of this project, with the purpose of assessing alcohol intoxication levels among spectators at football matches in the Swedish Premier Football League (SPFL). Specific research questions include: What is the proportion of spectators drinking alcohol? What is the average BAC level among the spectators? What is the proportion of spectators with a high level of intoxication, defined as having a BAC level of ≥ 0.1%? Which factors are predictive of a higher BAC level among the spectators?

## Materials and methods

### Design and setting

The current study used a cross-sectional design. Data were collected in Stockholm, the largest city and capital of Sweden, and Gothenburg, Sweden’s second largest city. The setting was all arenas (*n* = 3) located in Stockholm and Gothenburg hosting SPFL matches. These arenas are the homes to four SPFL teams, have the capacity for 18 000–50 000 spectators, and are located near the city centres of Stockholm and Gothenburg, respectively.

Inside the arenas, alcohol can be purchased at different Licensed Premises (LPs). Medium strength beer (3.5% by volume) can be purchased at kiosks, and all types of alcohol, including strong beer (> 4.5% by volume) and hard liquor, can be purchased at bars and pubs. The number of kiosks and bars/pubs varies by arena and by the estimated number of spectators at a particular match, but can be up to 16 kiosks and 10 bars and pubs in total. In addition, there are VIP lounges, available to sponsors, where alcohol, including hard liquor, can be obtained. In order to reduce the risk of alcohol-related problems at football matches, the Swedish Elite Football Association (SEFA), which is in charge of the SPFL together with the football teams, does not schedule matches on Friday or Saturday nights, but on weekday afternoons or evenings, and in the daytime or afternoon on weekends. At some high-risk matches such as local derbies (i.e., matches between two rival teams from the same city), only medium strength beer is available for purchase.

For safety reasons, the arenas are divided into different sections, e.g., the public section, the family section, and supporter sections for the home and away teams.

### Participants

Participants were recruited among spectators at the football arenas referred to above. The following inclusion criteria were used: a) being a spectator inside the arena and b) being 16 years or older. In total, 6132 spectators were approached and invited to participate in the study, and 4425 (72.2%) accepted the invitation. Among these, we excluded five individuals who were 15 years or younger. Thus, the total sample of the current study comprised 4420 spectators.

### Measurements

Data on demographics and recent self-reported alcohol use were assessed through a brief face-to-face interview developed by STAD. The interview comprised questions on gender, age, and alcohol use prior to having entered the arena. The time and location of the assessment was also noted. The level of alcohol intoxication was measured through the use of a breath analyser (Dräger Alcotest 6820, Drägerwerk AG & Co. KGaA, Germany), with the standardized sampling technique. This model is used by Swedish police authorities and older versions have been used in previous research [33].

### Procedure

Prior to data collection, all football clubs concerned and SEFA were contacted to gain approval for conducting BAC measurements during matches. The three arenas included in the study were visited on at least one occasion, in order to get an overview of the arenas and to decide on specific locations for the data collection.

Data were collected before and during SPFL matches which could take place on either weekdays (afternoon or evenings) or weekends (daytime or afternoon). At each event, staff from STAD and 18 research staff met about two hours before match kick-off. The research staff was divided into six data collection teams; thus, each team comprised three staff members, where one was in charge of recruiting participants, and the other two were in charge of conducting the interviews and collecting BAC data, respectively. It was decided that all recruiters should have an outgoing personality and be socially skilled, to facilitate inclusion of participants in the study.

The teams were placed at the aforementioned different sections of the arena to ensure generalizability. Data collection began approximately one hour before kick-off and was ongoing during the first half of the match, during the half-time break, and about five minutes into the second half of the match. Spectators were randomly selected and invited for study participation. The recruiter used a protocol where every third person who crossed an imaginary line, defined by each research team, was approached and invited to participate in the study. If the approached person belonged to a group, all members of the group were invited to participate, in order to reduce refusal rates [34,35]. If a person or a group of individuals declined to participate, they were recorded as drop-outs and gender(s) and estimated age(s) was/were noted by the team.

A glass of water was provided to each participant in order to clear the mouth cavity from alcohol and other debris. The brief face-to-face interview was then initiated, followed by the alcohol breath test. After completion of the test, which took approximately one minute, the BAC level was available and shown to the participant. If a participant expressed any concerns regarding his/her alcohol use, he or she was handed a written brochure on where to find further information and receive support. When the data collection was completed, all staff gathered to complete a protocol on reasons for drop-outs and how data collection went.

Data were collected at twelve football matches in total (four at each arena), which took place during the time period between April 17, 2015 and September 17, 2015. Among the twelve matches, three were local derbies. All research staff participated in a two-hour training session prior to data collection.

### Statistical analyses

Descriptive statistics, i.e., means, standard deviations (*SD*), frequencies and ranges, were computed to describe participants and settings. In addition, chi square-tests were used to test differences in proportions, while t-tests for independent samples and one-way analyses of variance (ANOVA) with Tukey post hoc-tests were used to compare means. In order to explore which factors were predictive of a higher BAC level, a multiple linear regression model was computed. In this model, gender, mean age, being a spectator at a local derby, day of assessment (i.e., weekday or weekend), self-reported alcohol use prior to having entered the arena, and sections of assessment (i.e., supporter sections or other sections) served as independent variables, while the average BAC level served as the dependent variable. As we had no specific hypotheses about the order or importance of the independent variables in relation to the dependent variable, they were entered simultaneously [36]. Prior to computing the analysis, data were checked for intercorrelations. The intercorrelations between independent variables in regression models should not exceed 0.70 and none did so in our data set. All data were analysed using SPSS version 23. *P* values < 0.05 were considered statistically significant.

### Ethics statements

All participants were verbally presented with information about study participation and an informed consent statement. Due to the importance of maintaining confidentiality, no signatures were collected and oral informed consent was used. The consent was documented on the notepad used during the face-to face interview. Ethical permission for the study, including the use of oral consent, was granted by The Regional Ethical Review Board at Karolinska Institutet in Stockholm (registration no. 2015-554-32-4).

## Results

### Sample characteristics

The overall study sample comprised 4420 participants. Among these, 82.4% were men 16.7% were women, and 0.9% (i.e. 39 participants) had not reported their gender (see Table 1). The mean age was 37.8 years. Participants assessed at Arena 1 had a higher mean age than participants assessed at Arena 2 (*F* (2,4419) = 3.340, *p* = 0.036. In the total sample, 47.8% reported alcohol use prior to having entered the arena (data missing for 29 participants). A larger proportion of participants drinking alcohol prior to having entered the arena was assessed at Arena 1 (59.0%) than at Arena 2 (37.0%) or Arena 3 (39.7%) (*X*^*2*^(2, 4420) = 199.653, *p* < 0.001). Furthermore, 31.2% of the spectators were assessed at a local derby, among which a larger proportion was assessed at Arena 1 (43.9%) than at Arena 2 (36.9%) (X^*2*^(1, 3351) = 15.828, *p* < 0.001). No BAC measurements were performed during local derbies at Arena 3.

**Table 1:** Participant characteristics of the overall sample and subsamples at each arena. Means (*M*), standard deviations (*SD*), frequencies, ranges and *p*-values for comparative statistics shown.

Variables	Overall sample (*n* = 4420)	Arena 1 (*n* = 2038)	Arena 2 (*n* = 1313)	Arena 3 (*n* = 1069)	*p*
Gender, % (*n*)					0.142
Men	82.4 (3643)	83.5 (1702)	82.4 (1073)	81.8 (868)	
Women	16.7 (738)	15.5 (316)	17.6 (229)	18.2 (193)	
Mean age, *M* (*SD*, range)					
Total sample	37.8 (13.7, 16–94)	38.1 (13.7, 16–94)	37.0 (13.9, 16–85)	38.1 (13.1, 16–87)	0.036 (1>2)
Participants reporting drinking prior to having entered the arena, % (*n*)	47.8 (2112)	59.0 (1202)	37.0 (486)	39.7 (424)	< 0.001
Participants assessed at a local derby, % (*n*)	31.2 (1379)	43.9 (894)	36.9 (485)	-	< 0.001
Participants with BAC > 0.0%, % (*n*)	46.8 (2068)	58.2 (1187)	36.6 (480)	37.5 (401)	< 0.001
Participants with BAC ≥ 0.1%, % *(n*)	8.9 (395)	11.8 (241)	6.3 (83)	6.6 (71)	< 0.001
Day of assessment, % (*n*)					< 0.001
Weekday	50.9 (2248)	41.0 (835)	67.3 (884)	49.5 (529)	
Weekend	49.1 (2172)	59.0 (1203)	32.7 (429)	50.5 (540)	
Sections of assessment, % (*n*)					< 0.001
Supporter sections	40.4 (1787)	31.6 (643)	61.6 (809)	31.3 (335)	
Other sections	59.6.(2633)	68.4 (1395)	38.4 (504)	68.7 (734)	

The proportion of spectators with BAC > 0.0% was 46.8% among all participants. A larger proportion of participants with BAC > 0.0% was assessed at Arena 1 (58.2%), than at Arena 2 (36.6% or Arena 3 (37.5%) (*X*^*2*^(2, 4420) = 199.563, *p* < 0.001). In the overall sample, 8.9% had a BAC level ≥ 0.1%, indicating a high level of intoxication. Here too, a larger proportion of the intoxicated spectators was assessed at Arena 1 (11.8%) than at Arena 2 (6.3%) or Arena 3 (6.6%) (*X*^*2*^(2, 4420) = 38.850, *p* < 0.001).

Approximately half (50.9%) of the total sample attended a weekday match (i.e., Monday, Wednesday, or Friday) or a weekend match (49.1% i.e. Saturday or Sunday). Additionally, 40.4% were assessed at supporter sections. A larger proportion of the BAC assessments during weekday matches took place at Arena 2 (67.3%) than at Arena 1 (41.0%) or Arena 3 (49.5%) (X^*2*^(2, 4420) = 222.995, *p* < 0.001), while the proportion of assessments performed at supporter sections was highest at Arena 2 (61.6%, Arena 1: 31.6% and Arena 3: 31.3%) (X^*2*^(2, 4420) = 348.079, *p* < 0.001). There was no significant difference in response rate between spectators at supporter sections or other sections of the arena (*X*^*2*^(1, 6132) = 3.133, *p* = 0.077).

### Average BAC levels among spectators

Average BAC levels among spectators are shown in Table 2. As demonstrated, the mean BAC level among participants with BAC > 0.0% was 0.063%. Among participants with ≥ 0.1%, the mean BAC level was 0.135%. Participants attending local derbies had a higher average BAC level (*M* = 0.066%) than participants attending non-derbies (*M* = 0.061%) (*t* (2066) = 2.537, *p* = 0.011), and spectators at supporter sections (*M* = 0.071%) had a higher mean BAC level than spectators at other sections (*M* = 0.056%) (*t* (2066) = 8.063, *p* < 0.001). The average BAC level at non-derbies was higher among participants at Arena 1 (*M* = 0.063%) than participants at Arena 2 (*M* = 0.054%), (*F* (2, 1260) = 3.735, *p* = 0.024). Also, the average BAC level at supporter sections was higher among participants at Arena 1 (*M* = 0.078%) than participants at Arena 2 (*M* = 0.064%) (*F* (2, 930) = 8.424, *p* < 0.001). No other differences in average BAC level were found between the participants at the three arenas.

**Table 2:** Mean Blood Alcohol Concentration (BAC) levels in the overall sample and subsamples at each arena. Means (*M*), standard deviations (*SD*), frequencies, ranges and *p*-values for comparative statistics shown.

Variables	Overall sample (*n* = 4420)	Arena 1 (*n* = 2038)	Arena 2 (*n* = 1313)	Arena 3 (*n* = 1069)	*p*
BAC level among participants with BAC > 0.0%, *M* (*SD*, range)					
Men and women	0.063 (0.044, 0.001–0.263)	0.064 (0.046, 0.001–0.263)	0.061 (0.043, 0.001–0.238)	0.061 (0.043, 0.006–0.226)	0.189
Men	0.063 (0.045, 0.001–0.263)	0.065 (0.046, 0.002–0.263)	0.062 (0.043, 0.001–0.238)	0.061 (0.042, 0.006–0.226)	0.345
Women	0.059 (0.042, 0.006–0.253)	0.063 (0.042, 0.006–0.253)	0.051 (0.040, 0.006–0.204)	0.056 (0.043, 0.006–0.143)	0.170
BAC level among participants with BAC ≥ 0.1%, *M* (*SD*, range)	0.135 (0.032, 0.100–0.263)	0.136 (0.033, 0.100–0.263)	0.134 (0.030, 0.100–0.238)	0.132 (0.029, 0.100–0.226)	0.657
BAC level among participants at local derbies with BAC > 0.0%, *M* (*SD*, range)	0.066 (0.045, 0.006–0.238)	0.065 (0.045, 0.006–0.234)	0.067 (0.045, 0.006–0.238)	-	0.591
BAC level among participants at non-derbies with BAC > 0.0%, *M* (*SD*, range)	0.061 (0.044, 0.001–0.263)	0.063 (0.047, 0.001–0.263)	0.054 (0.040, 0.001–0.218)	0.061 (0.042, 0.006–0.226)	0.024 (1>2)
BAC level among participants at supporter sections with BAC > 0.0%, *M* (*SD*, range)	0.071 (0.047, 0.001–0.258)	0.078 (0.048, 0.001–0.258)	0.064 (0.045, 0.060–0.238)	0.068 (0.045, 0.060–0.226)	< 0.001 (1>2)
BAC level among participants at other sections with BAC > 0.0%, *M* (*SD*, range)	0.056 (0.041, 0.001–0.263)	0.056 (0.043, 0.002–0.263)	0.053 (0.039, 0.001–0.204)	0.055 (0.039, 0.006–0.188)	0.688

### Prediction of higher BAC level among spectators

The results from the multiple linear regression model are presented in Table 3. A total of 68 individuals were excluded due to missing data in the independent variables, thus the analysis comprised 4352 participants. As shown, all independent variables entered in the model were associated with a higher BAC level. More specifically, male gender (*p =* 0.005), lower age (*p* < 0.001), attending a local derby (*p* < 0.001), self-reported alcohol use prior to having entered the arena (*p <* 0.001), attending a weekend match (i.e. Saturday or Sunday) (*p* < 0.001), and being a spectator at supporter sections (*p <* 0.001), significantly predicted a higher BAC level. The regression model was statistically significant (*F* (6, 4351) = 519.924, *p* < 0.001), with an R square of 0.42, showing that its independent variables explained 42% of the variance in the outcome.

**Table 3:** Prediction of mean Blood Alcohol Concentration (BAC) level by linear multiple regression. Unstandardized beta coefficients (B), standard errors (SE), standardized beta coefficients (β), *p-*values and 95% confidence intervals (CI) shown (*n* = 4352).

Variables	B	*SE*	*β*	*p*	95% CI Lower	95% CI Upper
Gender (male vs. female)	0.038	0.014	0.033	0.005	0.011	0.065
Age	-0.002	0.000	-0.049	< 0.001	-0.002	-0.001
Attending a local derby	0.053	0.011	0.057	< 0.001	0.031	0.076
Day of assessment (weekday vs. weekend)	-0.058	0.011	-0.067	< 0.001	-0.079	-0.038
Self-reported alcohol use prior to having entered the arena	0.529	0.010	0.607	< 0.001	0.508	0.549
Sections of assessment (supporter sections vs. other sections)	0.087	0.011	0.098	< 0.001	0.066	0.108
R^2^ = 0.42						

## Discussion

The current study aimed to assess the level of alcohol intoxication among spectators at matches in the SPFL. Nearly half of the participants had been drinking alcohol according to the BAC assessments, and the average BAC level among these individuals was 0.063%. Furthermore, the proportion of spectators with a high level of intoxication, defined as having a BAC level of ≥ 0.1% was 8.9%, among whom the average BAC level was 0.135%.

Our results have a number of implications. The average BAC level of 0.063% was higher than the BAC level of 0.057% reported from a previous study of spectators at baseball and football matches in the US [18]. A study of intoxication levels at licensed premises in the Swedish nightlife setting reported an average BAC level of 0.074% [37]. However, the setting of the current study differs substantially from the nightlife setting. For example, the BAC levels were collected at SPFL matches during the afternoon or daytime on weekends and during the afternoon or evening on weekdays, whereas the BAC levels of the aforementioned study were collected at licensed premises during late evenings or nights on weekends, and are thus expected to be much higher. Furthermore, the SPFL matches are family events where children are welcome, as opposed to licensed premises in the nightlife setting, where children are not welcome. Taken together, and in relation to the results from the study referred to above [37], the intoxication levels during matches in the SPFL can be considered to be rather high.

Moreover, about nine percent of the spectators had a high level of alcohol intoxication, defined as a BAC level ≥ 0.1%. Effects of such an intoxication level include, for instance, impairment of balance and motion, emotional instability and poor cognitive functioning [38]. Our results imply that there might be thousands of spectators with a high level of alcohol intoxication at SPFL matches. In line with previous research [15,16], they suggest that interventions are needed to reduce the alcohol intoxication at Swedish football matches.

Furthermore, male gender, lower age, attending a local derby, self-reported alcohol use prior to having entered the arena, attending a weekend match, and being a spectator at a supporter section were factors that were predictive of a higher BAC level. The aforementioned study by Glassman and colleagues also found that male spectators had a higher BAC level than female spectators, but there was no significant difference in such levels between older and younger spectators [20]. However, in the study by Ericsson and colleagues, younger age predicted an elevated BAC level, whereas male gender did not [18]. Furthermore, in contrast to our results, spectators attending matches at Monday nights were more likely to have a positive BAC level, than spectators attending matches at other nights. This may be explained by the fact that Monday Night Football (referring to American football) in the US is a particularly important event. In comparison to our study, the study by Ericsson and co-workers had a smaller sample size, and included spectators at both baseball and football matches, which also might contribute to the contrasting results. Our results, however, confirm findings of previous research demonstrating high levels of alcohol use in conjunction with derbies and football matches played during weekends [39–41]. Additionally, they provide information which may be used to predict which spectators have elevated BAC levels at football matches and to adjust interventions accordingly.

There are some limitations to this study. The conclusions that can be drawn from the results are limited due to the cross-sectional study design. Thus, the relationships between the independent variables and the dependent variable should be regarded as correlational rather than causal. Another limitation concerns the potential inclusion of intoxicated spectators with impaired cognitive functioning or judgment, contributing to biased responses during the interviews. Furthermore, based on a recommendation from SEFA and the fact that spectators tend to leave sporting events quickly, providing very little time for participant recruitment, data were collected prior to and during the matches, as opposed to the aforementioned US study [18], where data was collected at the end of or after the matches. Given that the use of alcohol might have been ongoing until the end of the match, our data collection procedure could hypothetically have produced lower BAC levels than would have been obtained if data had also been collected at the end of or after the matches. Our BAC levels may therefore have been underestimated, which can be considered to be a limitation of the study. Additionally, the current study assessed BAC levels at football matches in Sweden. Therefore, the results should not be generalized to other sporting events in other countries.

Some strengths should also be mentioned. The intoxication levels were assessed using biological sampling, not self-reported measures of alcohol use. Thus, our data are not biased by under- or over-reporting, and therefore, the validity of the results can be considered to be improved. Also, all research staff were given formal training prior to data collection, increasing the reliability of the results.

Another strength is the high response rate of approximately 72%. Furthermore, the research teams were placed at various sections of the arena, facilitating recruitment of individuals from the entire arena. Additionally, we used a standardized technique for participant recruitment which has been used in previous research [34,35]. Thus, we believe that we were able to obtain a representative sample of individuals who attend football matches in the SPFL, at least in the larger cities of Sweden. Finally, to our knowledge, this study is the first to assess alcohol intoxication through biological sampling among spectators at Swedish football matches, comprising a sample of over 4000 spectators. Thus, our study should make a strong contribution to the research field on intoxication levels among spectators at sporting events and factors that predict a higher BAC level at these events.

As highlighted by media and research, there is a need to reduce alcohol-related problems at sporting events in both Sweden and other countries. One strategy to fulfil this goal is to reduce the overall level of intoxication among spectators. As mentioned above, interventions developed by STAD have been found to be effective in reducing alcohol-related problems in the nightlife setting [24,26,28]. Due to the high intoxication levels obtained in this study, our research group is now planning to develop and implement a number of promising environmental prevention strategies such as community mobilization, RBS training to servers, training of security staff, policy work, media advocacy, and improved controls and sanctions. Whether these strategies result in lower intoxication levels at Swedish football matches will be examined. This study adds knowledge about the level of alcohol intoxication among spectators at sporting events and can inform researchers, public health officials, authorities, decision-makers, the sports community, and the general public.
